# Non-invasive global myocardial work index as a new surrogate of ventricular-arterial coupling in hypertensive patients with preserved left ventricular ejection fraction

**DOI:** 10.3389/fcvm.2022.958426

**Published:** 2022-09-23

**Authors:** Qin Duan, Hongmei Tao, Qian Dong, Kangla Liao, Yunjing Yang, Xiaocheng Cheng, Ping Ge

**Affiliations:** ^1^Department of Cardiology, The First Branch, The First Affiliated Hospital of Chongqing Medical University, Chognqing, China; ^2^Department of Cardiology, The First Affiliated Hospital of Chongqing Medical University, Chongqing, China; ^3^Department of Respiratory and Critical Care Medicine, The First Affiliated Hospital of Chongqing Medical University, Chongqing, China

**Keywords:** ventricular-arterial coupling, arterial hypertension, myocardial work, left ventricular performance, hypertension mediated organ damage

## Abstract

**Objective:**

As a new method of left ventricular-arterial coupling (VAC), the non-invasive myocardial work index (MWI) may provide more useful information than the classical methods of arterial elastance/left ventricular (LV) elastance index (the ratio of effective arterial elastance (Ea) over end-systolic elastance [Ea/Ees]). This research aims to investigate if MWI might be better associated with hypertension-mediated organ damage (HMOD) and diastolic dysfunction than Ea/Ees in hypertension.

**Methods:**

We prospectively enrolled 104 hypertensives and 69 normotensives. All subjects had speckle-tracking echocardiography for myocardial work, conventional echocardiography, and brachial-ankle pulse wave velocity (baPWV) measurements. The global work index (GWI) is a myocardial work component. The correlation between GWI and HMOD, as well as diastolic dysfunction, was analyzed. The receiver operating characteristic (ROC) curve was utilized for evaluating the GWI predicting efficacy.

**Results:**

The global work index was significantly higher in hypertensives than in normotensives (2,021.69 ± 348.02 vs. 1,757.45 ± 225.86 mmHg%, respectively, *p* < 0.001). Higher GWI was a risk factor on its own for increased baPWV, pulse pressure (PP), echocardiographic LV hypertrophy (LVH), and left atrial volume index (LAVI) (*p* = 0.030, *p* < 0.001, *p* = 0.018 *p* = 0.031, respectively), taking into account the sex, age, mean arterial pressure (MAP), body mass index (BMI), and antihypertensive therapy. However, no considerable associations were found between Ea/Ees and HMOD parameters and the diastolic dysfunction markers. The GWI area under the ROC curve for increased PP and baPWV, echocardiographic LVH, and increased LAVI were 0.799, 0.770, 0.674, and 0.679, respectively (*p* < 0.05).

**Conclusions:**

The global work index but not traditionally echocardiographic-derived Ea/Ees of VAC is independently related to HMOD and diastolic impairment in hypertensives with preserved LV ejection fraction. The GWI may be a potential marker for evaluating the VAC in hypertension.

## Introduction

Ventricular-arterial coupling (VAC) is defined by constant heart and arterial tree interaction, which reflects the global cardiovascular performance and has a pivotal function in the cardiac and aortic mechanics physiology (1).

Traditionally, VAC is most frequently assessed by echocardiography using the effective ratio of effective arterial elastance (Ea) over end-systolic elastance (Ea/Ees) (2). However, in hypertension, arterial stiffness may increase parallel with left ventricular (LV) myocardial stiffness, so the Ea/Ees ratio may comparatively stay stable, regardless of the fact that the stroke volume increased with an obvious systolic blood pressure increase (3). Thus, the Ea/Ees ratio benefit as a way to give more information about the ventricular-arterial system's physiologic and pathological status is limited. Although several adaptive alterations in the arterial tree and LV of hypertension are associated with the disease acuteness. Previous studies did not clearly show the linkage between the Ea/Ees ratio and hypertension-mediated organ damage (HMOD) and clinical outcomes (4, 5).

As the concept of VAC is evolving, the myocardial work index (MWI), derived using speckle tracking echocardiography from pressure-LV global longitudinal strain loop, is proposed as a novel VAC non-invasive method (6). Previous studies have shown it to be a sensitive index to quantify LV performance (7). In a recently published study of patients with hypertension, the MWI showed an increase against the raised afterload and a downtrend when hypertrophy and myocardial remodeling occur (8). Chan et al. researched 74 patients with hypertension and dilated cardiomyopathy and indicated that MWI was a useful tool to understand LV remodeling and increased wall stress correlation in various loading statuses (9). The current research was performed to further investigate whether MWI might be better linked to cardiac and vascular damage than Ea/Ees in hypertension.

## Methods

### Study population

**Figure 1:**
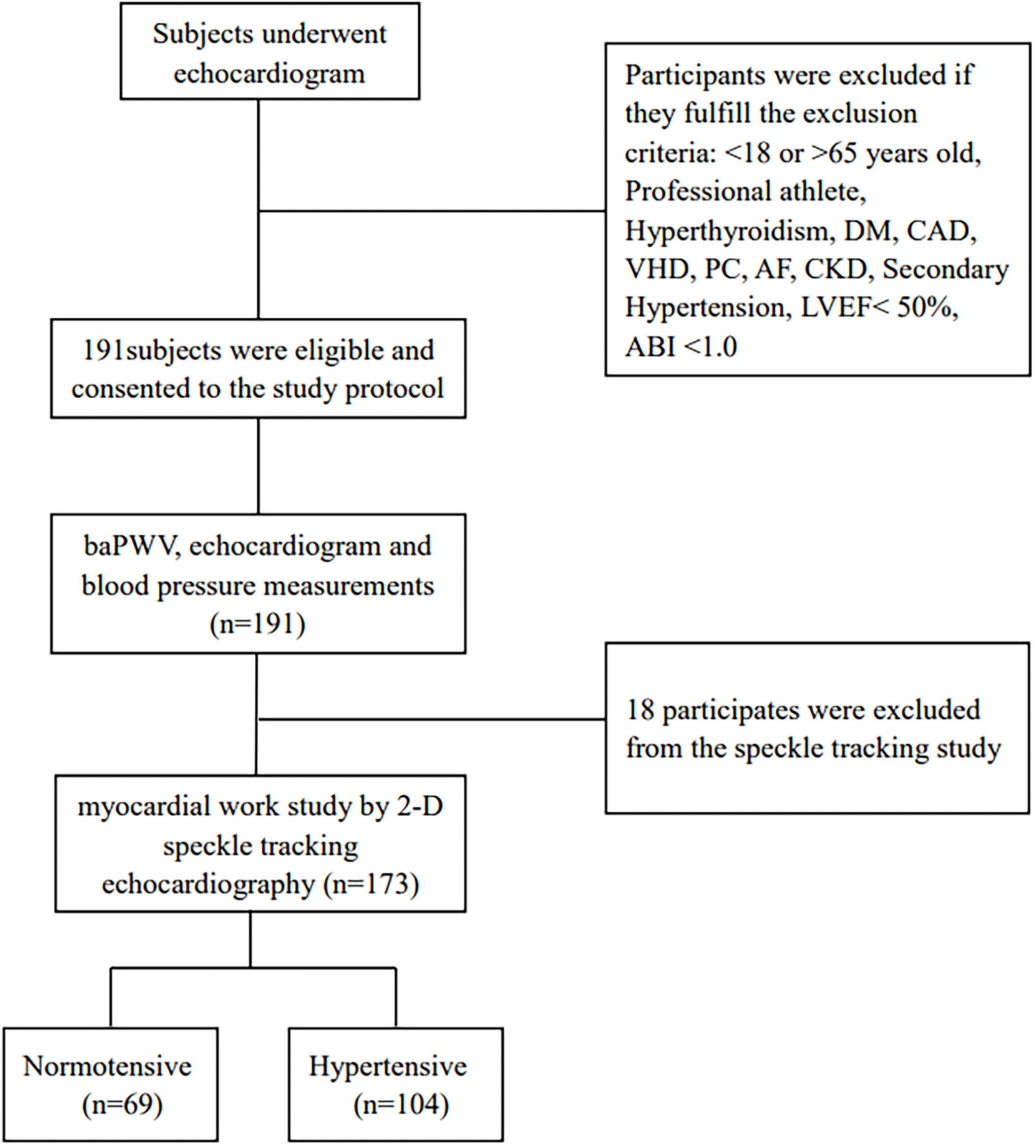
Flow diagram for the study population. DM, diabetes mellitus; CAD, coronary artery disease; VHD, valve heart disease; PC, primary cardiomyopathies; AF, arterial fibrillation; CKD, chronic kidney disease; LVEF, left ventricular ejection fraction; ABI, ankle-brachial pressure index.

The current research was prospective and single-centered, done between 14 September 2020 and 30 December 2020 at the echocardiography center of the First Affiliate Hospital of Chongqing Medical University, Chongqing, China. It comprised consecutive normotensive and hypertensive participants ranging from 18 to 65 years old with LVEF ≥ 50%. According to 2018, ESC/ESH guidelines for the management of arterial hypertension, the systolic and/or diastolic blood pressure of ≥ 140 mmHg, or ≥ 90 mmHg, respectively, were used to define hypertension, as well as any antihypertensive medication usage, or both (10). The systolic or diastolic blood pressure of <130 or < 85mmHg, respectively, were used to define normotension (10). The 1/3^*^SBP + 2/3^*^DBP formula was utilized to compute the MAP. The pulse pressure (PP) was derived by subtracting diastolic pressure from systolic pressure. According to the established protocol, each individual had a comprehensive clinical assessment, such as hypertension history, blood pressure, weight, height, waist, smoking, and alcohol use status, electrocardiograph, and brachial-ankle pulse wave velocity (baPWV). Professional athletes and individuals with documented diabetes mellitus, chronic kidney dysfunction, atrial fibrillation, hyperthyroidism, valvular heart disease, coronary artery disease or its symptoms, secondary hypertension, ankle-brachial pressure index (ABI) of < 1, and primary cardiomyopathies were excluded. This research initially included 191 subjects by excluding 18 individuals with suboptimal echocardiographic images, and finally, 104 hypertensives and 69 normotensives underwent LV myocardial work and strain assessment using the 2D speckle-tracking echocardiography method (Figure 1).

The institutional ethics committee approved this research (approval No. 2020-606), following the “Declaration of Helsinki.” On clinicaltrials.gov, the research was registered (approval No. NCT04573257). All individuals gave consent to participate.

### Echocardiography examination

The Vivid E95, a commercially available system, was utilized in the study. The American Society of Echocardiography instructions were followed in the study recordings and measurements (11). Doppler, speckle tracking deformation imaging and conventional 2D were utilized to assess all the individuals. The software (GE Medical Systems, version 203.88) was utilized to store and analyze the standard echocardiography 2D images of the cardiac cycles. The linear method was utilized to determine the LV mass. LV mass index (LVMI) was calculated as followes: LVMI = LV mass/body surface area. By the sex-dependent cut-off values application, LV hypertrophy was identified (12): LVMI > 115 and >95 g/m^2^ for men and women, respectively. The Simpson biplane and area-length methods were applied to compute the Left atrial and LV volumes. The E velocity was measured by applying the pulse wave Doppler sample volume at the mitral valve tips. The tissue Doppler imaging (TDI) was used to record the early diastolic septal and lateral annular e'velocities.

The Ea and Ees were expressed as ESP divided by stroke volume (SV) (ESP/SV) and ESP divided by end-systolic volume, respectively. The ESP equals brachial systolic blood pressure multiplied by 0.90. The Ea/Ees ratio was then simplified to ESV/SV (13).

The following four variables and their abnormal cut-off values were utilized to evaluate the LV diastolic dysfunction: average E/e' ratio > 14, septal annular e' velocity < 7 cm/s, peak tricuspid regurgitation (TR) velocity > 2.8 m/s, and left atrial volume index (LAVI) > 34 ml/m^2^ (14).

The methodology to analyze myocardial work and LV global longitudinal strain (GLS) was validated in previous publications (15, 16); briefly, at the LV end-systole, the endocardial apical 4, 2, and 3 chamber borders views were manually traced. Automatically, the longitudinal strain curves were processed, and the GLS was determined as the average value of 18 segments across the three views. LV myocardial work (MV) was determined by the GLS and brachial artery blood pressure; first, the GLS was computed, then the mitral and aortic valves opening and closing were timed, and lastly, the brachial blood pressure was determined. The four components of MV were global constructed work (GCW), global wasted work (GWW), global work index (GWI), and global work efficiency (GWE). The work index was the pressure–strain loop area; wasted work was the work done while systole lengthening and isovolumetric relaxation shortening; constructive work was the work done while systole shortening and isovolumetric relaxation lengthening; and work efficiency was computed by dividing the constructive work over the constructive and wasted work summation. The GWW, GCW, GWI, and GWE were the average values of 18 segments across the three views. These assessments were executed by one trained and experienced observer blinded to clinical and demographic data. Intra-observer agreement for the analysis was very good (correlation coefficient = 0.93, *p* < 0.001).

### BaPWV and ABI

The baPWV was measured with a commercially available PWV/ABI device (Omron Colin BP-203RPE III). The baPWV measurement was conducted on the same day as echocardiography. For a minimum of 5 min of rest, the individual was first positioned supine in rest, and then around the bilateral upper arms and ankle, four blood pressure cuffs were put before connecting to oscillometric and plethysmographic pressure sensors. At the bilateral brachia and ankles, the devices record the arterial blood pressure, volume pulse form, and phonogram. The distance from the ankle to the right brachium was measured. Automatically, by dividing the transmission distance over the transmission duration, the baPWV was derived. ABI was determined bilaterally by calculating the ankle-SBP to brachium-SBP ratio on both sides. For analysis, the mean baPWV and ABI for both sides were utilized.

### Statistical analysis

As mean ± standard deviation (SD) or median and interquartile range, the continuous variables were presented as per the Kolmogorov–Smirnov test for distribution normality, and as frequencies and percentages, the categorical variables were presented. The statistically considerable differences were observed by the Mann–Whitney test or the unpaired student's *t*-test for normally distributed variables otherwise. The GWI and Ea/Ees correlates were evaluated by Spearman's or Pearson's correlation analysis according to the distribution of the variables. The binary logistic regression was utilized to define the VAC markers and their components (GWI, Ea, Ees, Ea/Ees ratio) with HMOD indices and LV diastolic dysfunction, such as (1) elevated or normal arterial stiffening [baPWV > 1,400 cm/s vs. baPWV ≤ 1,400 cm/s (17), and PP ≥ 60 mmHg vs. PP < 60 mmHg (10)]; (2) echocardiographic LVH or not (LVMI > 115 and > 95 g/m^2^ for men and women, respectively vs. LVMI ≤ 115 and ≤ 95 g/m^2^ for men and women, respectively) (10); (3) impaired or normal markers of LV diastolic function (septal e' velocity < 7 cm/sec vs. septal e' velocity ≥ 7 cm/sec, average E/e' ratio > 14 vs. average E/e' ratio ≤ 14 and LAVI > 34 ml/m^2^ vs. LAVI ≤ 34 ml/m^2^) (14). Sex, age, MAP, BMI, and antihypertensive therapy were corrected by the forward multiple regression analysis. The 95% confidence interval and the estimated odds ratio were computed. The SPSS version 19.0 statistical software was utilized for all analyses. The two-sided *p*-values of < 0.05 were regarded as statistically significant.

## Results

### All participants' baseline characteristics

The hypertensive individuals' median age was 50.00 (44.25–56.75) years. These patients have a median SBP of 144.00 (135.00–154.00) mmHg, median DBP of 90.50 (84.25–97.75) mmHg, and MAP of 107.67 (101.33–115.58) mmHg. The median history of hypertension in these patients was 2.00 (1.00–5.00) years. Table 1 reveals the baseline features, including the study population's echocardiographic VAC and demographic data. Hypertensive and normotensive participants had similar age, fractional shortening (FS), heart rate (HR), LV ejection fraction (LVEF), CI (cardiac index), LAVI, and Ea/Ees, whereas other indicators including baPWV, stroke volume (SV), LVMI, GWI, GLS, average E/e' et al. differed significantly between the two groups. In comparison with the normotensive group, Ea and Ees increased parallel for hypertensives, and Ea/Ees ratio was finally the same in the two groups (0.56 (0.50–0.63) vs. 0.55 (0.51–0.59), respectively, *p* = 0.577). GWI was considerably higher in hypertensives than in normotensives (2,021.69 ± 348.02 mmHg% vs. 1,757.45 ± 225.86 mmHg%, respectively, *p* < 0.001). In both groups, no considerable correlation was noted between GWI and Ea/Ees.

**Table 1:** Baseline characteristics of the study population.

**Variable**	**Overall (*n* = 173)**	**Control group (*n* = 69)**	**Hypertensive (*n* = 104)**	***p***
**Demographic data**				
Age (years)	49.00 (43.00–56.00)	48.00 (38.50–54.00)	50.00 (44.25–56.75)	0.102
Male/Female	94/79	26/43	68/36	<0.001
SBP (mmHg)	132.00 (118.00–147.50)	116.00 (108.50–121.00)	144.00 (135.00–154.00)	<0.00
DBP (mmHg)	82.00 (76.00–93.00)	74.00 (68.00–81.00)	90.50 (84.25–97.75)	<0.001
MAP (mmHg)	100.67 (89.33–109.50)	88.00 (81.50–92.33)	107.67 (101.33–115.58)	<0.001
HR (bpm)	76.16 (64.60–84.76)	75.05 (64.38–85.28)	76.58 (66.42–83.67)	0.564
BMI (kg/m^2^)	24.42 (22.15–26.91)	22.83 (21.12–25.01)	25.33 (23.60–27.47)	<0.001
Waist (cm)	84.88 ± 9.97	80.42 ± 9.00	87.79 ± 9.53	<0.001
Current smoking	36 (20.8%)	12 (17.4%)	34 (32.7%)	0.026
Current drinking	36 (20.8%)	12 (17.40%)	34 (32.7%)	0.026
LVH in UCG	50 (28.9%)	5 (7.2%)	45 (43.3%)	<0.001
baPWV	1,493.50 (1,328.00–1,715.00)	1,310.50 (1,220.00–1,465.25)	1,615.00 (1,469.00–1,827.5)	<0.001
ABI	1.12 ± 0.07	1.08 ± 0.07	1.13 ± 0.07	<0.001
**Standard echocardiographic data**				
IVST (mm)	10.00 (9.00–11.00)	9.00 (9.00–10.00)	11.00 (10.00–12.00)	<0.001
PWT (mm)	10.00(9.00–11.00)	9.00 (8.00–10.00)	11.00 (9.00–12.00)	<0.001
LVEDD (mm)	46.00 (43.00–48.00)	45.00 (42.00–47.00)	47.00 (44.00–49.00)	0.001
LVESD (mm)	30.00 (28.00–31.00)	29.00 (27.00–30.00)	30.00 (28.25–32.00)	<0.001
FS (%)	35.00 (33.00–37.00)	35.00 (34.00–36.75)	35.00 (35.00–37.00)	0.562
LVEF (%)	64.00 (62.00–67.00)	64.50 (63.00–66.00)	64.00 (61.00–67.00)	0.559
CI (L/min/m^2^)	2.75 ± 0.61	2.70 ± 0.65	2.78 ± 0.56	0.404
SV (ml)	61.69 ± 11.72	57.97 ± 9.96	64.24 ± 12.08	<0.001
LVMI (g/m^2^)	97.47 ± 21.95	83.01 ±15.49	106.37 ± 21.11	<0.001
LAVI (ml/m^2^)	24.93(21.57–29.55)	25.46 (21.29–28.74)	25.14(22.13–31.00)	0.378
Average E/e'	8.86 ± 2.23	7.98 ± 2.00	9.53 ± 2.18	<0.001
e' Septum	6.45 (5.50–8.90)	7.8 (6.35–9.50)	6.10 5.00–7.60)	<0.001
**Myocardial work and strain data**				
GWI (mmHg%)	1,912.66 ± 328.43	1,757.45 ± 225.86	2,021.69 ± 348.02	<0.001
GWE (%)	95.00 (93.00–96.00)	96.00 (94.00–97.00)	94.00 (91.25–96.00)	<0.001
GCW (mmHg%)	2,315.90 ± 359.02	2,116.88 ± 228.59	2,454.30 ± 371.31	<0.001
GWW (mmHg%)	100.50 (64.25–163.00)	79.00 (52.50–106.50)	128.00 (84.25–204.75)	<0.001
GLS (%)	19.64 ± 2.09	20.57 ± 1.71	19.03 ± 2.10	<0.001
**Ventricular–arterial coupling data**				
Ea (mmHg/mL)	1.92 (1.69–2.21)	1.79 (1.62–2.00)	2.08 (1.79–2.37)	<0.001
Ees (mmHg/mL)	3.58 ± 0.85	3.29 ± 0.60	3.76 ± 0.934	<0.001
Ea/ Ees	0.55(0.50–0.61)	0.55 (0.51–0.59)	0.56(0.50–0.63)	0.577

SBP, systolic blood pressure; DBP, diastolic blood pressure; MAP, mean arterial pressure; HR, heart rate; BMI, body mass index; LVH, left ventricular hypertrophy; UCG, ultrasound cardiogram; baPWV, brachial-ankle pulse wave velocity; ABI, ankle brachial index; IVST, interventricular septal wall thickness; PWT, posterior wall thickness; LVEDD, left ventricular end-diastolic dimension; LVESD, left ventricular end-systolic dimension; FS, fractional shortening; LVEF, left ventricular ejection fraction; CI, cardiac index; SV, stroke volume; LVMI, left ventricular mass index; LAVI, left atrial volume index; GWI, global work index; GWE, global work efficiency; GCW, global constructed work; GWW, global wasted work; GLS, global longitude strain; Ea, effective arterial elastance; Ees, end-systolic elastance.

Female subjects, as compared to male subjects in hypertensives, had higher GWI values (2,233.03 ± 326.06 mmHg% vs. 1,909.81 ± 306.52 mmHg%, respectively, *p* < 0.001), however, the GWI values were similar in older and younger patients by utilizing the median age (50 years) as a cut-off.

### VAC index in normotensive subgroup

Supplementary Table S1 revealed the relationship between GWI and Ea/Ees with major clinical and echocardiographic parameters in normotensive subjects. GWI was related with SBP, MAP, PP, GLS (*r* = 0.516, *p* < 0.001; *r* = 0.380, *p* = 0.001; *r* = 0.450, *p* < 0.001; *r* = 0.629, *p* < 0.001; respectively) and with LAVI (*r* = 0.449, *p* < 0.001). No considerable correlation was noted between Ea/Ees index and SBP, MAP, PP, LVMI, baPWV, and LV diastolic function markers except for a significant correlation of the Ea/Ees index with LVEF and GLS (*r* = −0.971, *p* < 0.001 and *r* = −0.268, *p* = 0.026).

### Ventricular-arterial coupling indexes and HMOD and LV diastolic dysfunction in hypertension

**Figure 2:**
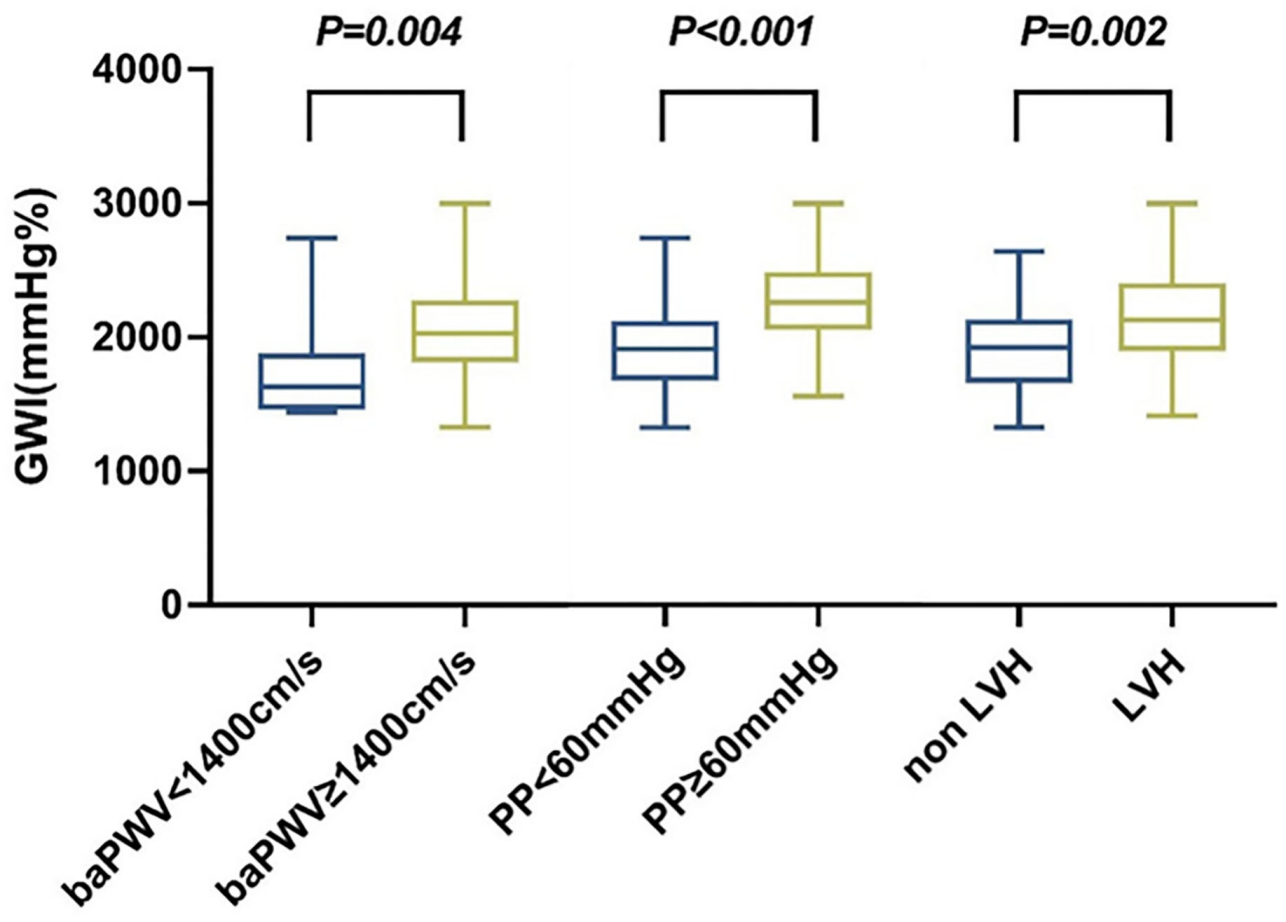
Distribution of GWI in hypertension subjects according to cut-offs of baPWV, PP, and LVH. GWI, global work index; BaPWV, brachial-ankle pulse wave velocity; PP, pulse pressure; LVH, left ventricular hypertrophy.

Hypertension-mediated organ damage indexes in the study included baPWV > 1,400 cm/s, PP ≥ 60 mmHg, and echocardiographic LVH. Increased baPWV, PP, and echocardiographic LVH were detected in 93, 45, and 32, respectively, hypertensive subjects. The distribution of GWI in hypertension subjects according to the cut-off of baPWV, PP, and LVH is shown in Figure 2. Patients with increased baPWV and PP and echocardiographic LVH had higher GWI (*p* = 0.004, *p* < 0.001, *p* = 0.002, respectively).

As shown in Table 2, in simple binary logistic regression, GWI was considerably linked to baPWV > 1,400 cm/s (*p* = 0.023), PP ≥ 60 mmHg (*p* = 0.023) and echocardiographic LVH (*p* = 0.002). By multivariate analysis and adjusting for the sex, age, MAP, BMI, and antihypertensive therapy, GWI was still an independent risk factor of baPWV > 1,400 cm/s, PP ≥ 60 mmHg, and echocardiographic LVH (*p* = 0.030, *p* < 0.001, *p* = 0.018, respectively). However, no considerable correlations were observed between Ea/Ees and baPWV > 1,400 cm/s, PP ≥ 60 mmHg, and echocardiographic LVH.

**Table 2:** Association of global work index, Ea/Ees and its components with hypertension-mediated organ damage end points.

	**Odds ratio**	**95%CI**	***p***
**baPWV** **>** **1,400 cm/s**			
Univarite			
GWI	1.003	1.000–1.005	0.023
Ea/ Ees	4.92	0.004–6,607.22	0.665
Ea	22.14	2.36–207.98	0.007
Ees	2.86	1.11–7.37	0.029
Multivarite			
**GWI corrected for age, sex, MAP, BMI and antihypertensive therapy**			
GWI	1.002	1.000–1.005	0.030
**PP** **≥60 mmHg**			
Univarite			
GWI	1.004	1.002–1.005	0.023
Ea/ Ees	6.477	0.062–675.80	0.431
Ea	2.50	1.001–6.230	0.050
Ees	1.29	0.831–2.014	0.254
Multivarite			
**GWI corrected for age, sex MAP, BMI and antihypertensive therapy**			
GWI	1.005	1.003–1.007	<0.001
Sex (Femal vs. male)	3.912	1.122–13.64	0.032
**LVH by LVMI** **>115 g/m**^**2**^ **for men and** **>95 g/m**^**2**^ **for women**			
Univarite			
GWI	1.002	1.001–1.003	0.002
Ea/ Ees	0.250	0.003–20.094	0.536
Ea	0.848	0.378–1.900	0.688
Ees	0.934	0.614–1.421	0.751
Multivarite			
**GWI corrected for age, sex MAP,BMI and antihypertensive therapy**			
GWI	1.002	1.000–1.003	0.018
age	1.071	1.012–1.134	0.018
MAP	1.058	1.012–1.106	0.013
Antihypertensive therapy (yes vs. no)	0.286	0.286–0.102	0.017

baPWV, brachial-ankle pulse wave velocity; BMI, body mass index; GWI, global work index; Ea, effective arterial elastance; Ees, end-systolic elastance; MAP, mean arterial pressure; PP, pulse pressure; LVH, left ventricular hypertrophy; LVMI, left ventricular mass index.

Left ventricular diastolic function impaired markers in this study including septal e'velocity < 7 cm/s, average E/e' ratio > 14 and LAVI > 34 ml/m^2^ were detected in 70, 4, and 20, respectively, of hypertensive individuals. There was no patient with TR velocity > 2.8 m/s.

There was also an association of GWI with LAVI in the univariate logistic regression (*p* = 0.006), and this association was retained statistically significant in a multivariate model such as age, sex, MAP, BMI, and antihypertensive therapy (*p* = 0.031), as revealed in Table 3. However, no considerable association was noted between Ea/Ees and all these three LV diastolic function impaired markers.

**Table 3:** Association of global work index, Ea/Ees and its components with left ventricular diastolic dysfunction parameters.

	**Odds ratio**	**95%CI**	***p***
**e'septum** **<** **7 cm/sec**			
Univarite			
GWI	0.999	0.998–1.001	0.267
Ea/ Ees	0.063	0.001–6.234	0.238
Ea	0.980	0.425–2.261	0.963
Ees	1.161	0.738–1.827	0.518
**LAVI>34 ml/m** ^**2**^			
Univarite			
GWI	1.002	1.001–1.004	0.006
Ea/ Ees	0.162	0.001–44.733	0.526
Ea	0.740	0.251–2.185	0.586
Ees	0.907	0.529–1.553	0.722
Multivarite			
**GWI corrected for age, sex MAP, BMI and antihypertensive therapy**			
GWI	1.002	1.001–1.003	0.031
**Average E/e'** **>** **14**			
Univarite			
GWI	1.002	1.000–1.003	0.058
Ea/ Ees	2.973	0.004–2,297.512	0.748
Ea	2.494	0.857–7.258	0.093
Ees	1.511	0.829–2.753	0.178

BMI, body mass index; GWI, global work index; Ea, effective arterial elastance; Ees, end-systolic elastance; LAVI, left atrial volume index; MAP, mean arterial pressure.

**Figure 3:**
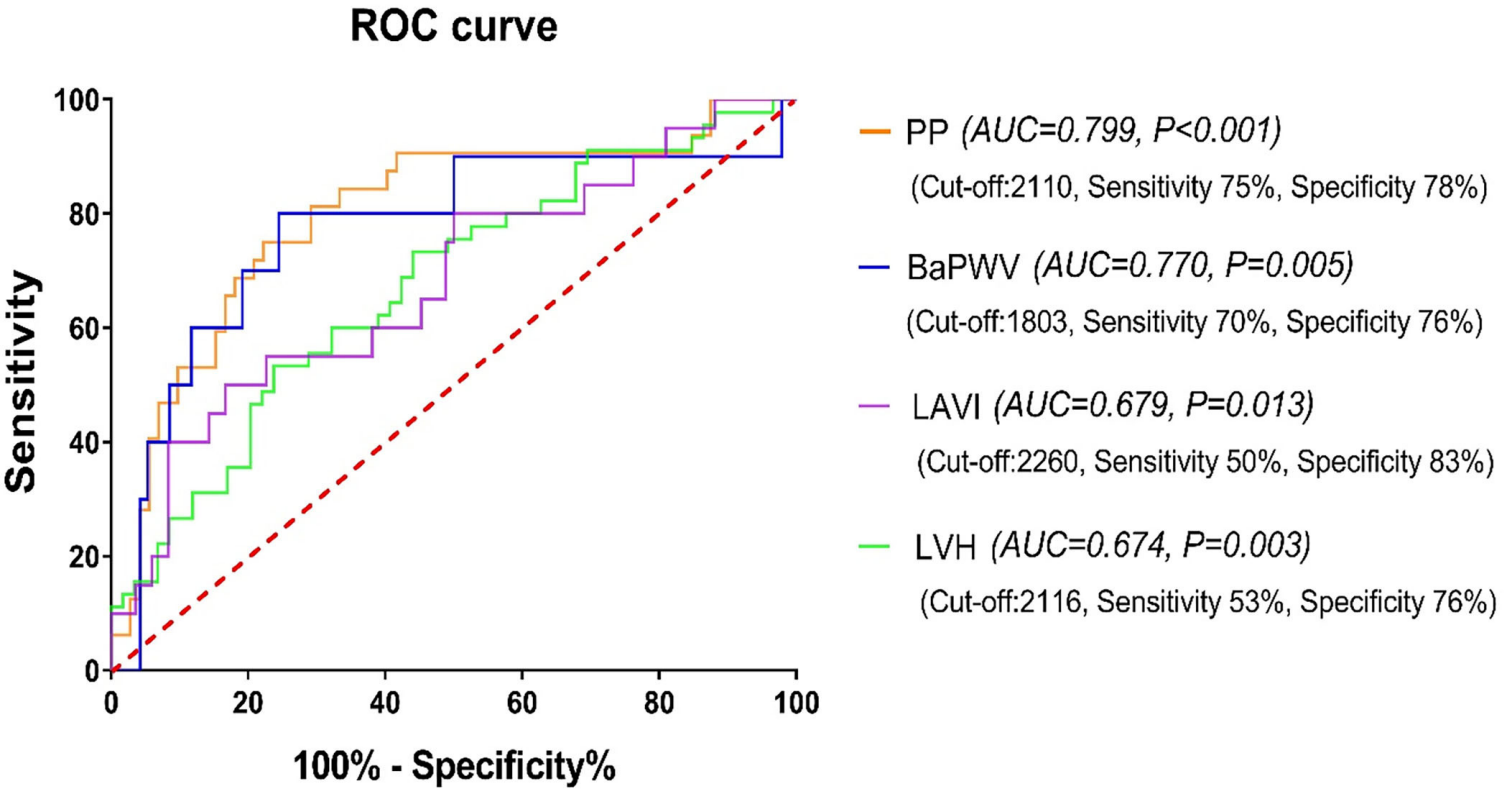
The receiver operating characteristic (ROC) curve of GWI for predicting HMOD and increased LAVI in the hypertensive subjects. GWI, global work index; HMOD, hypertension-mediated organ damage; LAVI, left atrial volume index; PP, pulse pressure; BaPWV, brachial-ankle pulse wave velocity; LVH, left ventricular hypertrophy.

The GWI area under the curve (*AUC*) to predict increased PP and baPWV, echocardiographic LVH, and increased LAVI was 0.799, 0.770, 0.674, and 0.679, respectively (all *p* < 0.05). The cut-off values were 2,110 mmHg% (sensitivity 75%, specificity 78%), 1,803 mmHg% (sensitivity 70%, specificity 76%), 2,260 mmHg% (sensitivity 50%, specificity 83%), and 2,116 mmHg% (sensitivity 53%, specificity 76%), respectively, as revealed in Figure 3.

## Discussion

The GWI and Ea/Ees were measured in a population of hypertensive and normotensive controls. The research's primary findings were (1) GWI was considerably elevated, but the Ea/Ees was similar in the hypertensives in comparison with the normotensives; and (2) GWI but not Ea/Ees in hypertensives was linked to HMOD, such as increased baPWV and PP and echocardiographic LVH, and also associated with increased LAVI, after adjusting for sex, age, and MAP.

The vessel and heart need to be considered as a unique system; thus, the assessment of cardiovascular performance should incorporate the examination of ventricular properties and the arterial system regulating effects. VAC refers to the heart-pumping action coupled with the arterial system load resistance, which can be easily described as the Ea/Ees ratio, and eventually determined the cardiovascular system performance and efficiency (18). It was a crucial hemodynamic evaluation element in severely ill patients (19) and provided a wider hemodynamic disorders perspective linked to prevalent conditions, such as heart failure (1, 20), septic shock (21), or right ventricular dysfunction (22). However, although the excellent pathophysiological background of Ea/Ees, the traditionally and frequently used echocardiography-derived Ea/Ees ratio method met some endogenous limitations in the clinical setting, especially in hypertension and heart failure with preserved ejection fraction (HFPEF) (23, 24). The pathologic changes of endothelial dysfunction and fibrosis in hypertension affect both arterial walls and myocardium, leading to the increase in the ventricular and arterial stiffening (25). This provided a relatively stable Ea/Ees and implied the shortcoming of Ea/Ees for early assessment of the cardiovascular function in hypertensives. In line with previous studies, no considerable differences were noted in Ea/Ees in hypertensive and normotensive groups in the present study. And neither HMOD indicators, such as increased baPWV, increased PP, and the existence of LVH nor dystoloic dysfunction indexes consisting of septal e' velocity < 7 cm/s average E/e' ratio > 14 and LAVI > 34 ml/m^2^ were founded to be associated with Ea/Ees ratio in hypertensives.

The global work index was calculated as the LV pressure–strain loop area. As a myocardial work component obtained from a non-invasive LV pressure–strain loop by speckle tracking echocardiography, it was considered a novel VAC marker that affects the myocardial oxygen metabolism and cardiovascular function (7). It has been examined in many cardiac diseases and exhibited a promising application in hypertension (8, 9, 26). As a compensatory strategy to maintain the LV function opposing the elevation in afterload, Chan et al. revealed that, in patients with moderate-to-severe hypertension, the GWI was considerably higher (9). Filip et al. further found that the GWI increased in acute pressure overload and decreased when myocardial remodeling and hypertrophy appears (8). In addition, the GWI was found to be higher in patients with uncontrolled and resistant hypertension (27). The current study further investigated the relationship between GWI and HMOD and diastolic dysfunction. In our study, hypertensives with increased baPWV, PP and LAVI, and echocardiographic LVH had significantly higher GWI. After adjusted parameters such as age, sex, MAP, body size, and antihypertensive therapy, which were known to influence VAC (3, 5, 28, 29), GWI was a risk factor on its own for increased baPWV, PP, LAVI, and echocardiographic LVH. This suggested that the subclinical disease progression with GWI early assessment might have a pivotal function for the linkage with asymptomatic target organ damage, such as arterial stiffening and echocardiographic LVH, and diastolic dysfunction detected by increased LAVI. In addition, further research is mandatory to determine if the interventions targeting to decrease GWI would benefit the HMOD and diastolic dysfunction in hypertension. Furthermore, although the indexes of HMOD involved in the study have been demonstrated to be associated with the prognosis in hypertensives (30–34), whether the GWI was associated with future cardiovascular events and mortality needs to be investigated to determine its clinical value as an indicator of VAC in hypertension.

Of note, although both GWI and Ea/Ees were indexes of VAC, no considerable GWI and Ea/Ees correlation was noted in the hypertensive group, as well as in normotensives, probably owing to the different properties of these two methods.

There are some limitations of this study. First, not all parameters of asymptomatic HMOD were included as it was an exploratory research that aimed to indicate the GWI fundamental clinical value. Second, this research was based in one location, and the cross-sectional aspect did not conclude real causation between GWI and HMOD, such as arterial stiffness and LVH and increased LAVI. Third, the echocardiographic-derived Ea/Ees limitations in the present study, rather than using the gold standard estimate of Ea/Ees from the invasively obtained pressure–volume curve, might partly account for the significant Ea/Ees with HMOD and diastolic dysfunction association. Fourth, patients' hypertension is usually complicated by diabetes, coronary heart disease, and other conditions, but these factors were excluded in this study in order to exclude the influence of these factors on myocardial work and HMOD. Therefore, the results of this study are not applicable to all patients with hypertension. Moreover, we evaluated the arterial stiffening by baPWV but not by the gold standard methodology of carotid-femoral pulse wave velocity (cfPWV) (35). Nevertheless, baPWV has been widely investigated and demonstrated as a good index of arterial stiffening to predict future cardiovascular events and mortality (34).

In summary, in patients with hypertension, the GWI but not the echocardiographic-derived Ea/Ees ratio of VAC is related to HMOD, including arterial stiffening and LVH, and diastolic impairment evaluated by increased LAVI. The GWI may be a new potential marker for the VAC assessment and, finally the early cardiovascular performance in hypertension.

## Data availability statement

The data analyzed in this study is subject to the following licenses/restrictions: The datasets generated and/or analyzed during the current study are not publicly available due to limitations of ethical approval involving the patient data and anonymity but are available from the corresponding author on reasonable request. Requests to access these datasets should be directed to geping216022@163.com.

## Ethics statement

The studies involving human participants were reviewed and approved by First Affiliate Hospital of Chongqing Medical University, Chongqing, China. All individuals gave consent to participate.

## Author contributions

The research was undertaken by QDu, HT, QDo, KL, and YY. Data analysis was performed by QDu and XC. The manuscript was written by QDu and HT. The study design and the manuscript review were undertaken by QDu, PG, and XC. All authors reviewed the manuscript.

## Funding

This study was supported by Chongqing Science and Technology Commission, key project, (2019 ZLXM003); Chongqing Science and Technology Commission, Youth Project (2018QNXM024).

## Conflict of interest

The authors declare that the research was conducted in the absence of any commercial or financial relationships that could be construed as a potential conflict of interest.

## Publisher's note

All claims expressed in this article are solely those of the authors and do not necessarily represent those of their affiliated organizations, or those of the publisher, the editors and the reviewers. Any product that may be evaluated in this article, or claim that may be made by its manufacturer, is not guaranteed or endorsed by the publisher.
