# Supplementary material for: Non-invasive global myocardial work index as a new surrogate of ventricular-arterial coupling in hypertensive patients with preserved left ventricular ejection fraction
Source: Front Cardiovasc Med. 2022 Sep 23;9:958426. doi: 10.3389/fcvm.2022.958426 (PMC9543298; doi:10.3389/fcvm.2022.958426)
Supplement: Supplementary file 1 [file Table_1.docx]

| Variable | GWI | |  | Ea/Ees | |  |
| --- | --- | --- | --- | --- | --- | --- |
|  | coefficient | *P* |  | coefficient | *P* |  |
| Age(years) | 0.128 | 0.296 |  | 0.169 | 0.165 |  |
| BMI(kg/m^2^) | 0.003 | 0.983 |  | 0.157 | 0.196 |  |
| SBP(mmHg) | 0.516 | <0.001 |  | 0.048 | 0.698 |  |
| MAP(mmHg) | 0.380 | 0.001 |  | 0.043 | 0.727 |  |
| PP(mmHg) | 0.450 | <0.001 |  | 0.087 | 0.479 |  |
| baPWV(cm/s) | 0.078 | 0.525 |  | 0.133 | 0.276 |  |
| LVMI(g/m^2^) | 0.142 | 0.246 |  | 0.021 | 0.864 |  |
| GLS(%) | 0.629 | <0.001 |  | -0.268 | 0.026 |  |
| LVEF(%) | 0.022 | 0.857 |  | -0.971 | <0.001 |  |
| LAVI (ml/m^2^) | 0.449 | <0.001 |  | 0.000 | 0.997 |  |
| e’ Septum(cm/s) | 0.081 | 0.508 |  | -0.075 | 0.543 |  |
| Average E/e’ | 0.218 | 0.084 |  | -0.053 | 0.679 |  |

Supplementary table S1. Correlation of GWI and Ea/Ees with major clinical and echocardiographic parameters in normotensive subjects.

GWI, global work index; Ea/Ees, the ratio of effective arterial elastance (Ea) over end-systolic elastance (Ees); BMI, body mass index; SBP, systolic blood pressure; MAP, mean arterial pressure; PP, pulse pressure; baPWV, brachial-ankle pulse wave velocity; LVMI, left ventricular mass index; GLS, global longitude strain; LVEF, left ventricular ejection fraction; LAVI, left atrial volume index.
